# Land Cover and Topography Affect the Land Transformation Caused by Wind Facilities

**DOI:** 10.1371/journal.pone.0088914

**Published:** 2014-02-18

**Authors:** Jay E. Diffendorfer, Roger W. Compton

**Affiliations:** U.S. Geological Survey, Geosciences and Environmental Change Science Center, Denver Federal Center, Denver, Colorado, United States of America; Plymouth University, United Kingdom

## Abstract

Land transformation (ha of surface disturbance/MW) associated with wind facilities shows wide variation in its reported values. In addition, no studies have attempted to explain the variation across facilities. We digitized land transformation at 39 wind facilities using high resolution aerial imagery. We then modeled the effects of turbine size, configuration, land cover, and topography on the levels of land transformation at three spatial scales. The scales included strings (turbines with intervening roads only), sites (strings with roads connecting them, buried cables and other infrastructure), and entire facilities (sites and the roads or transmission lines connecting them to existing infrastructure). An information theoretic modeling approach indicated land cover and topography were well-supported variables affecting land transformation, but not turbine size or configuration. Tilled landscapes, despite larger distances between turbines, had lower average land transformation, while facilities in forested landscapes generally had the highest land transformation. At site and string scales, flat topographies had the lowest land transformation, while facilities on mesas had the largest. The results indicate the landscape in which the facilities are placed affects the levels of land transformation associated with wind energy. This creates opportunities for optimizing wind energy production while minimizing land cover change. In addition, the results indicate forecasting the impacts of wind energy on land transformation should include the geographic variables affecting land transformation reported here.

## Introduction

Wind energy is one of the fastest growing segments of the electricity market in many nations and this trend will likely continue as countries strive to reduce greenhouse gas emissions while meeting growing energy demands [1]. Research continues to predict the environmental consequences of new wind generation [2]–[4], compare impacts of different energy production technologies [5], consider methods for optimal placement of wind facilities [6], [7] and study the role wind should play in future energy strategies [8]–[11]. All of these activities require fundamental information that relates wind installations to the energy they produce and their impacts (both positive and negative) on economic, cultural, and environmental systems.

One impact of wind facilities is land transformation caused by building and maintaining the facility. These changes to land surface, including roads, can create management concerns for species affected by habitat loss and fragmentation [12]. We follow Fthenakis and Kim [5] use of “land transformation” and use ha/MW to define it following Denholm et al. [13]. Thus, land transformation describes the amount of land transformed to produce one MW of capacity.

To date, studies of wind power utilize estimates of land transformation for GIS-based modeling [4] or basic calculations of the land area required to generate energy using wind [9], [14]. However, published estimates vary by more than 1000 times (0.06 to 135 ha/MW, Table 1) with the estimated amount of area needed to meet the United States Department of Energy's land-based wind production goal of 251GW [9] ranging from ∼151 to 338,000 km^2^. These levels of uncertainty make analyses using point estimates subject to scrutiny and comparisons to other forms of energy extremely difficult.

**Table 1:** Reported values of land transformation associated with wind development, the original value transformed into ha/MW, and the predicted amount of land transformation required to meet 251GW of land based wind energy—a stated goal of the Department of Energy (DOE) [9].

Studies	Reported value	ha/MW	km^2^ for 251GW
Roads and infrastructure only			
Denholm et al. [13] (low)	0.06 ha/MW	0.06	151
BLM [23] (low)	0.4 ha/1.5 MW turbine	0.27	678
Denholm et al. [13] (mean)	0.3 ha/MW	0.3	753
DOE [9] (low)	2% of 5MW/km2	0.4	1,004
BLM [23] (high)	1.2 ha/1.5 MW turbine	0.8	2,008
DOE[9](high)	5% of 5MW/km2	1	2,510
Denholm et al. [13] (high)	2.4 ha/MW	2.4	6,024
Entire project area			
Denholm et al. [13] (low)	4.76 ha/MW	4.76	11,948
DOE [9]	5 MW/km2	20	50,200
Denholm et al. [13] (mean)	34.5 ha/MW	34.5	86,595
Elliot [25] (high)	2.65 MW/km2	37.7	94,717
Mackay [26]	2W/m2	50	125,500
Elliot (low)	1.03 MW/km2	97.1	243,689
Pimentel et al. [3]	1 billion kwh/yr/13700ha	120.1	301,683
Denholm et al. [13](high)	135 ha/MW	135	338,850

The reported values are organized based on the lands directly transformed by roads and infrastructure development and the entire project area. Some studies reported ranges of values and means, labeled as “low,” “mean,” and “high.”

Why do these estimates vary so much? Different methodologies explain some of the variation in reported land transformation. For example, methods including just the direct land transformation of a facility produce much lower estimates than methods that define the area as a polygon around the facility or the land leased by the facility (Table 1). However, even within each of these general approaches, estimates of land transformation vary substantially. We note that *none* of the studies in Table 1 actually measured surface disturbance from working wind facilities using modern GIS-based approaches. Instead, calculations often assumed a relationship between turbines and surface disturbance, used planning documents to estimate disturbance, or made assumptions about turbine spacing and land requirements.

Another explanation is that factors such as turbine size, topography, and pre-development land cover affect the land transformation caused by wind facilities. All of these variables could influence the distance between turbines, the amount of new roads, and the land cleared to install and operate a facility. For example, Denholm et al. [13], in perhaps the most thorough study to date, estimated land transformation from 172 environmental impact reports of wind facilities and reported average values by: turbine string configuration, land cover, and the total nameplate capacity of the facility. The reported mean values suggested land transformation of wind facilities were influenced by geographic variables and string configuration though no formal statistical analyses were performed.

Table 1 indicates the land transformation associated with wind facilities is perhaps poorly measured and not well understood. Understanding factors affecting land transformation will make future forecasts of the spatial impacts of wind power more accurate. Furthermore, if geographic features such as topography and land cover affect the land transformation caused by wind facilities, decision makers can use this knowledge to plan facilities that maximize energy production while minimizing surface impacts.

We report results from a geospatial analysis of 39 wind facilities we fully digitized using high resolution photo-imagery. We selected the facilities and designed our analyses to elucidate the effects of turbine size, turbine string configuration, topography, and land cover on land transformation. The results indicate the high levels of variation in land transformation across facilities can be explained, in part, by geographic variables.

## Materials and Methods

We digitized wind facilities in the United States selected to span gradients in turbine size, land cover, topography, and string configuration. We used turbine size information for each facility from the Energy Information Agency (EIA) to categorize facilities into four turbine size classes (<1.5, 1.5 to <2.0, 2.0 to <2.5, and >2.5 MW). When we began the work, turbine location data were not publicly available, as it is now, from the Federal Aviation Agency. We searched for facilities based on turbine size and the limited locational data in the EIA database, and other sources (Google Earth, the websites of wind energy companies, and county-level information). When we found a location, we digitized turbine locations using 1-m resolution USDA/NRCS Digital Orthophoto Quad Imagery (DOQ) county mosaics (http://datagateway.nrcs.usda.gov/) and then verified our turbine counts with those in the EIA database. We also verified facilities by matching photographs from company websites with aerial imagery, measuring turbine blades to verify make and model of turbines, matching this to facility information, and contacting county agencies for siting information when necessary.

Facilities were categorized using visual interpretation of aerial imagery. Land cover included categories describing both the general vegetation type and human use of the land. The categories were forest, shrub, grassland, hay, and tilled. Hay locations were mowed but not tilled, whereas grasslands were naturally occurring grasslands typically used for grazing. Topography included simple categories of flat, hills, ridgelines, and mesas. Flat locations had almost no topographic relief, whereas areas with turbines across numerous hills were categorized as hills. In many cases turbines were placed to follow the edge of a ridge or cliff. We categorized these as ridgeline. Some facilities were placed on mesas, an elevated area of land with a flat top and steep cliffs. When the turbines only followed the cliff edge, we considered these ridgelines, but when turbines were along both the cliff edge and across the top of the mesa, we considered these mesas. See File S1 for examples of facility classifications. We followed Denholm et al. [13] and categorized the configuration of turbines at a facility as single string, parallel strings, multiple strings, or clustered. Multiple string facilities included more than one string of turbines but these were not in parallel lines whereas clustered facilities had turbines scattered across an area with no obvious strings.

Finding facilities across all combinations of explanatory variables was impossible. For example, facilities with 3MW turbines in both flat and forested sites simply did not exist. As such, we could not design a fully orthogonal study. Instead, we balanced the objective to sample across the categories described above with the difficulty of finding and verifying facilities and our budget. In addition, we avoided using multiple phases of development or closely spaced facilities as replicates. This process reduced issues related to pseudoreplication, but also limited our sample sizes in some cases. Though RWC digitized nearly 17,000 turbines, we ultimately could identify and use 39 facilities. Table 2 describes sample sizes by categories used in the analyses.

**Table 2:** Sample sizes associated with each categorical variable used in the analyses.

Variable	Category within variable				
Land use/cover	Forest	Grassland	Hay	Shrub	Tilled
	7	5	8	9	10
Topography	Flat	Hills	Ridgelines	Mesa	
	15	4	13	7	
Turbine Size	<1.5 MW	1.5–<2.0 MW	2–<2.5 MW	>2.5 MW	
	9	12	11	7	
String Configuration	Clustered	Multiple	Parallel	Single	
	6	15	6	12	

In all cases, total sample is 39.

### Digitizing procedures

We focused on estimating *new* land transformation caused by the development of a wind facility, not all of the infrastructure necessary to install, run, and maintain a facility. We did this for two reasons. First, we could not confidently understand the complete set of infrastructure required to support a facility from aerial photography, particularly if some infrastructure did not change during the installation of the facility. Second, we wanted to understand and quantify if placing facilities in areas with preexisting infrastructure, such as a robust road network, affected the amount of new infrastructure required.

Knowing the installation date of each facility, we compared ortho-imagery prior to, and after installation to determine the land transformation. We digitized any land transformation caused by the facility installation including new or widened roads, gravel pits, staging and storage areas, communication or meteorological towers, turbine pads and string roads, buried cables, disturbance to the original landscape caused by landslides, road berms, or erosion control treatments, roads and vegetation clearing associated with aerial transmission, and electricity substations (File S1).

We approached land transformation mapping conservatively. For example, in cases where vegetation recovered rapidly, or in agricultural areas where buried cables did not affect future farming activities, we did not include these short-lived disturbances. We did not include the original disturbance associated with the facility if vegetation had recovered (based on aerial imagery) within five years or if the original land cover (farming) was still viable (File S1, Slide 1). In general, this only happened in tilled locations or in moist grasslands where grasses recovered rapidly. We also treated roads conservatively, by subtracting preexisting roads from new or widened roads when the two overlapped. Finally, we only digitized surface disturbance if it could be linked specifically to the wind facility (File S1, Slide 2). For example, some facilities shared an electricity substation with other facilities, or road networks linked different phases of facility development. When questions arose regarding what facility caused a specific transformation, we always erred conservatively and did not include the surface change in our digitizing.

### Statistical analysis

The facilities we digitized were highly variable in their structure. Some had extremely long access roads, buried cables, or transmission lines that generally connected the facility to existing roads or power lines. These idiosyncratic connecting infrastructures made large contributions to a facilities land transformation. To address this issue, we analyzed facilities at three spatial scales: “strings,” “sites,” and entire “facilities.” “Strings” represented the smallest spatial unit and consisted of turbine pads and the roads and buried cables connecting them. Strings allowed us to analyze relationships between turbine size and land transformation without the addition of jump roads between strings, gravel pits, and other forms of surface disturbance. “Sites” included the strings, the roads or electrical connections between strings, and other infrastructure associated with the strings. Long access roads or connections to the larger power grid were not included in sites. Finally, “facilities” included all digitized land transformation.

We estimated the total area of land transformation and the area attributed to roads for each facility at each spatial scale. To estimate land transformation, we divided the area of land we mapped as transformed (ha) by the facilities nameplate capacity (MW). We also estimated turbine spacing by calculating the average nearest-neighbor distance between turbine locations at a facility.

We use generalized linear models to analyze the effects of turbine size, land cover, topography, and string configuration on land transformation at each spatial scale. Unlike land transformation, turbine locations were fixed and did not vary with spatial scale, so analyses of the mean nearest-neighbor distance between turbines was only done once.

Generalized linear models are a class of statistical models that share a number of properties, such as linearity and computational approaches for estimating parameters [15]. Our generalized linear models were structured so that either land transformation or nearest-neighbor distance between turbines were the dependent variables, and combinations of turbine size, land cover, topography, and string configuration were included as categorical explanatory variables. The models included a Gaussian error distribution and an identity link function. Sample size was 39 facilities for all models whereas the number of parameters varied depending on the combination of explanatory variables included.

Because multiple explanatory variables may drive the land transformation of wind facilities simultaneously, our analyses were based on the approach of multiple working hypotheses [16], [17]. Model selection based on information theory is well suited for these types of analyses [18], and we followed well-described approaches [19]. We performed model selection by developing a list of candidate models that included both individual and additive combinations of explanatory variables then ranked models using Akaike's Information Criterion adjusted for finite sample sizes (AICc). Model selection was based on the differences in AICc across models where models within 2 AICc were considered to have substantial support. We also used AICc weights, which range from 0 to 1, and represent the weight of evidence that a model is the best model given the data and the set of candidate models. To understand the relative importance of explanatory variables we summed the AICc weights across all the models where the variable occurred. Larger values of the summed AICc weights indicate which explanatory variables most affected land transformation or turbine spacing [20]. We used model averaging to estimate effect sizes and report means and estimates of precision based on unconditional standard errors [19], [21]. All analyses were done in R [22].

## Results

Similar to previously reported values, land transformation varied considerably across facilities. The range declined from the facility scale (0.11–4.3 ha/MW) to the site (0.11–1.5 ha/MW) and string (0.11–1.3 ha/MW) scales illustrating how road and transmission lines that connect facilities to transportation networks and the electric grid can increase land transformation.

### Land transformation

Model selection indicated land cover and topography, but not turbine configuration or size, were generally associated with the amount of land transformation across facilities. Land cover or topography commonly occurred in the best-supported models and had the highest parameter AICc weights (Tables S1–S3). An exception was at the facility scale, where topography had low AICc weights and was not in any of the most supported models, whereas turbine size had higher AICc weights and was in the best-supported models (Table S3). This result was caused by the long access road and transmission line at Kibby Mountain, which also had large, 3MW turbines. At the facility scale, Kibby Mountain, with an extremely large land transformation, affected model rankings, enhancing the role of turbine size. Given this unique situation, we doubt this result is generalizable. At the other spatial scales (Tables S1 and S2) turbine size was not found in the best-supported models, and its variable weights were small, indicating little to no effects on land transformation.

**Figure 1:**
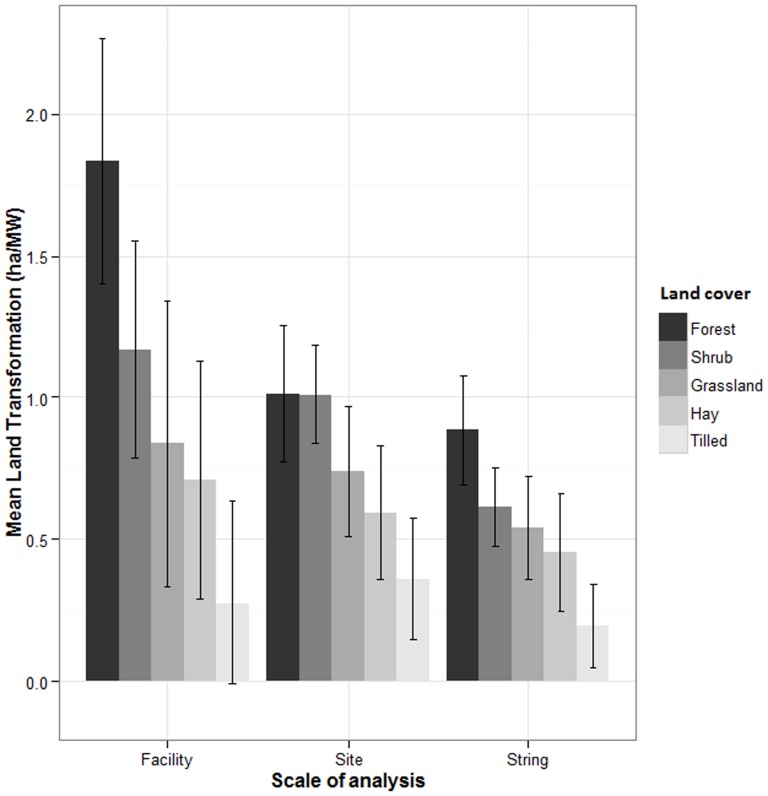
Mean (±95% Confidence Interval) of the land transformation associated with wind facilities in different land use and cover (“Land cover”) categories at 3 spatial scales of analysis.

**Figure 2:**
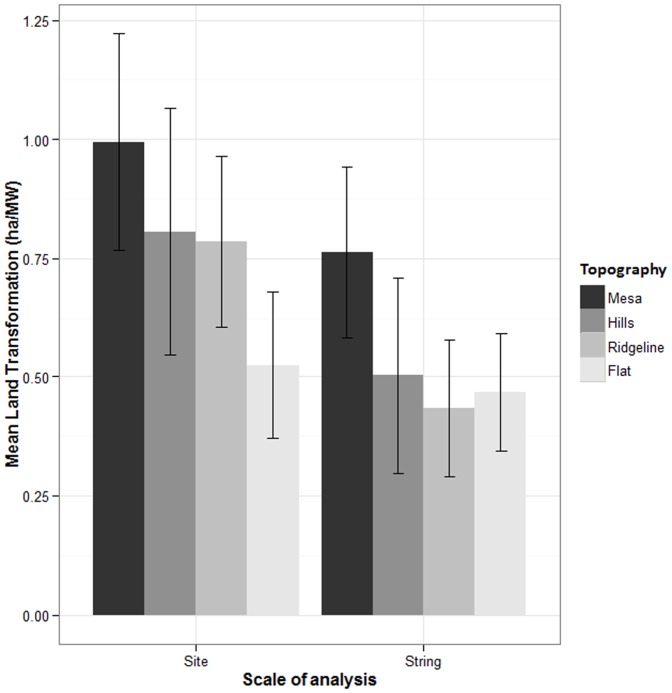
Mean (±95% Confidence Interval) of the land transformation associated with wind facilities in different topographic categories at 2 spatial scales of analysis.

At all spatial scales, facilities on tilled landscapes had lower levels of land transformation than forests and shrublands (Fig. 1). Facilities on mesas had higher land transformation values than facilities in flat topographies at the string and site scales (Fig. 2). Topography did not affect land transformation at the scale of entire facilities.

Mean land transformation at the facility (0.93±0.12 ha/MW, mean+SE) and the site scales (0.72±0.06 ha/MW) were two to three times higher than the mean value (0.3 ha/MW) derived from a review of environmental impact reports (Table 1, see Denholm et al. [13] for road and infrastructure only). At all scales, the surface of new roads (not berms or other disturbances associated with roads) accounted for 38–41% of a facilities' land transformation (facility scale: 41%±0.04, site scale: 38%±0.04, string scale: 41%±0.04, mean±SE).

### Turbine Spacing

**Figure 3:**
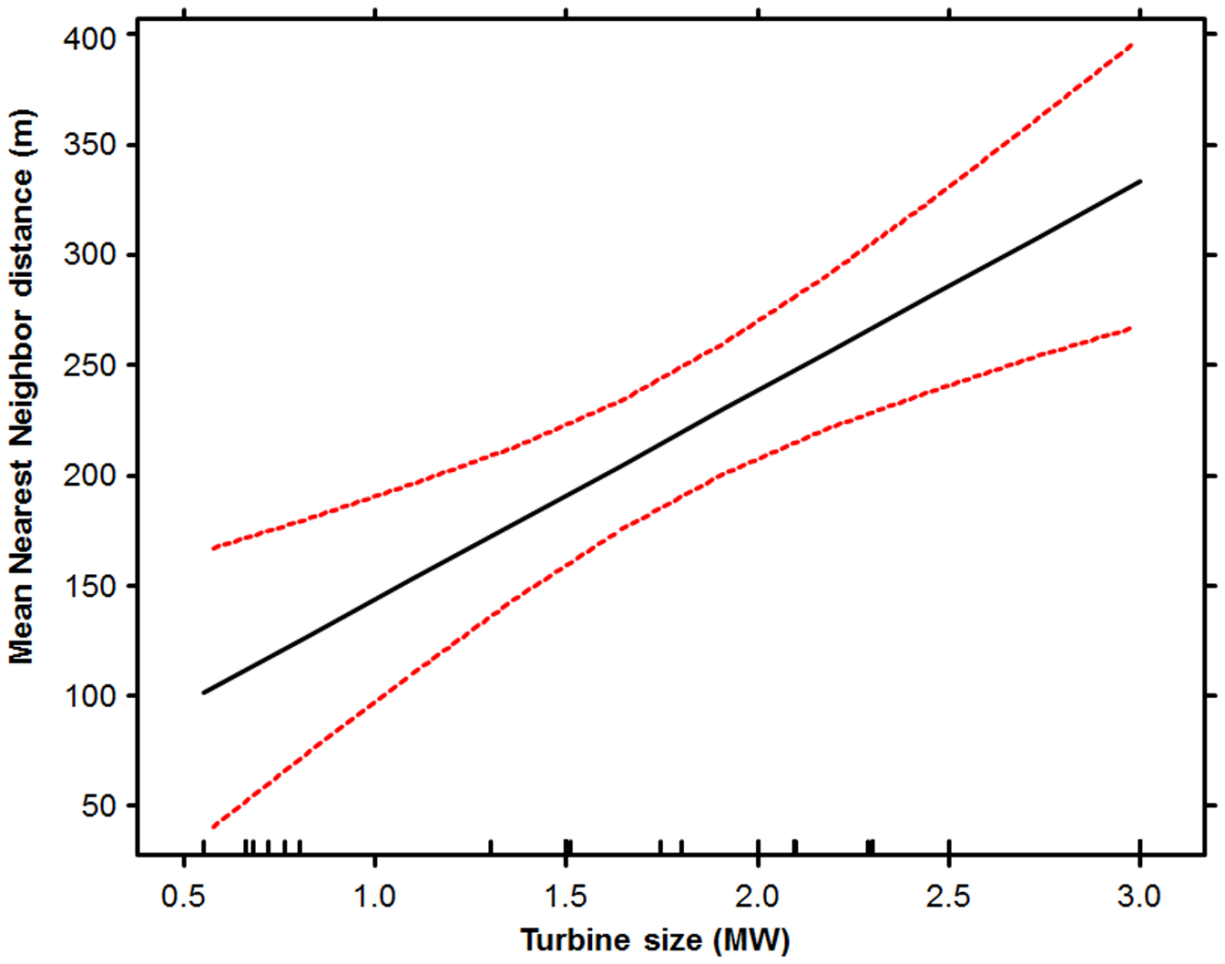
The linear relationship (black line) and 95% Confidence Interval (red lines) between the mean nearest neighbor distance among turbines at a facility and the size of the turbines in MW of capacity.

Land cover and turbine size affected turbine spacing. Tilled locations (357.7±48.2 m, mean ±95% confidence interval had higher turbine spacing than other types of land cover (Forest  = 226.7±60.3, Grassland  = 219.8±70.8, Hay  = 211.7±57.6, Shrub  = 211.32±53.7). In addition, larger turbines had higher distances between them (Fig. 3, Table S4).

## Discussion

The results indicate the levels of land transformation associated with wind energy vary substantially across facilities. Furthermore, geographic variables are more likely to drive levels of land transformation than turbine-specific variables. Where a facility was built affected the amount of land transformed more so than the size of the turbines or how they were configured. Furthermore, our statistical models produced estimates of effect sizes for those variables driving land transformation, allowing predictions of the land transformation associated with wind energy.

Wind facilities can change through time and space as turbines are added or removed. Our study represents a single snapshot of land transformation from industrial scale wind energy. The long connecting roads and transmission lines at some facilities had large impacts on their land transformation. For example, Kibby Mountain, with the highest land transformation at the facility scale (4.3 ha/MW) had a ∼47 km transmission line and a 14 km road (File S1, Slide 3). If these were installed to support multiple phases of development, then future estimates of land transformation at Kibby Mountain may decrease. We performed our analyses at three spatial scales in part because connecting infrastructure increased land transformation, yet the use of long roads and power lines appeared sporadically and with no apparent patterns across facilities. Despite this, land cover affected land transformation at all three spatial scales, whereas topography affected land transformation at the site and string scales.

At least two processes may explain why tilled landscapes had lower levels of land transformation. First, tilled landscapes have a large road network capable of supporting heavy agricultural machinery. Facilities in tilled landscapes use these existing roads, typically building a small road to connect an individual turbine to a preexisting road. Second, land was replanted in agricultural fields where cables were buried, as were road berms or small scrapes near the turbine pad (File S1, Slide 1). Other land cover types did not have the robust road networks, and surface disturbance associated with facility installation was not rapidly re-used.

Land transformation in forests was generally higher than other land cover types. At the facility scale, this was likely driven by the high land transformation associated with Kibby Mountain. However, even at the site and string scales, land transformation at facilities in forests was generally higher than other land cover types. Larger clearings around turbines, roads, and intersections caused by tree and canopy removal may drive this pattern (File S1, Slides 3 and 4). For example, trees must be cleared in wide arcs around intersection corners to make room for turbine blades on flatbed trucks. In addition, trees were cleared around pads and roads at what appeared to be distances long enough to avoid hazards associated with tree falls.

We expected topography to affect land transformation by requiring longer and more sinuous roads in areas with higher levels of relief. At the site scale, flat geographies had the lowest average land transformation. However, mesas were generally flat yet facilities on mesas had the highest average land transformation. We are uncertain of the underlying mechanism, but placing turbines along ridge edges in combination with additional turbines across tops of mesas, or other flat areas may require more complex road networks (File S1, Slide 5).

One might expect greater distances between turbines would require longer roads and hence more land transformation. However, tilled landscapes had the largest distances between turbines yet the smallest land transformation. Unlike larger tracts of land with parallel lines of turbines, turbines were frequently more scattered in agricultural areas. We suspect lease agreements with individual landowners and zoning laws such as road and residence setback requirements, may result in wider spacing between turbines in tilled landscapes relative to other locations.

The juxtaposition between wider turbine spacing yet less land transformation could influence decisions about the optimal location of new wind development. Our results suggest that for a fixed generating capacity, a facility in an agriculture landscape would require a greater amount of total area (if measured as a polygon around the turbines), yet produce less new land transformation relative to the same capacity in a different land-use type.

Our main argument is that geographic variation in land transformation exists and should be considered when forecasting or planning for wind energy, we hope that other researchers and policy makers will consider using more than a single point estimate of land transformation for wind energy. Our estimate for entire facilities (0.93 ha/MW) was higher than all but two previously reported values (1 ha/MW by the Bureau of Land Management (BLM) [23], and 2.4 ha/MW by Denholm [13], Table 1). The estimate from BLM was considered a high estimate, whereas the estimate from Denholm represented the maximum value in their data. Our highest values were 4.3 and 2.3 ha/MW. If one assumes direct measurements of land transformation are more accurate than estimates based on build out models, environmental impact reports, or derivations based on turbine spacing, then the majority of point estimates used in planning may have underestimated land transformation associated with new facilities.

Our work has implications for studies attempting to forecast future land transformation associated with wind development. Given the high levels of variation in land transformation reported here and elsewhere, using a single point estimate of land transformation for broad-scale generalization will likely produce vague and perhaps even misleading results, particularly when comparing land transformation across energy types. For example, using model-averaged coefficients for tilled landscapes to project new land transformation for 100GW of wind energy predicts 686 km^2^ whereas using coefficients for forests yields 4606 km^2^. We suggest using model coefficients that relate geographic variables to land transformation, in conjunction with geospatial map layers, to forecast land transformation from wind and other forms of energy production.

## Conclusions

Ultimately, our results suggest the geographic context where energy is developed will play a fundamental role in the levels of actual or forecasted impact. This makes comparisons across energy types difficult, as we must control for underlying differences in pre-development factors that could influence impacts such as land cover, quantities and qualities of water and biodiversity, or even the perceived aesthetic value of a location. Such analyses will become complex if the impacts for different energy types changes with the geographic setting. For example, wind energy benefits on health and CO_2_ reduction caused by displacing fossil fuels varies considerably across the U.S. [24]. Though adding complexity, variation across landscapes in variables that drive the impacts of energy development ultimately creates opportunities to plan national energy strategies that minimize impacts while maximizing benefits.

## Supporting Information

Table S1 **Model results for land transformation at the string scale.** Sum of variable AICc weights are: Land cover  = 0.99, Topography  = 0.46, Turbine size  = 0.17, Configuration  = 0.01. (DOC)

Table S2 **Model results for land transformation at the site scale.** Sum of AICc weights are: Land cover  = 0.99, Topography  = 0.74, Turbine size  = 0.18, Configuration  = 0.02. (DOC)

Table S3 **Model results for land transformation at the facility scale.** Sum of AICc weights are: Land cover  = 0.99, Topography  = 0.05, Turbine size  = 0.56, Configuration  = 0.11. (DOC)

Table S4 **Model results for the mean nearest neighbor distance between turbines.** Sum of AICc weights are: Land cover  = 0.97, Topography  = 0.03, Turbine size  = 0.99, Configuration  = 0.05. (DOC)

File S1 **Powerpoint file illustrating digitized wind turbine facilities in different land cover and topography categories.**
(PPTX)
