# Supplementary material for: Land Cover and Topography Affect the Land Transformation Caused by Wind Facilities
Source: PLoS One. 2014 Feb 18;9(2):e88914. doi: 10.1371/journal.pone.0088914 (PMC3928332; doi:10.1371/journal.pone.0088914)
Supplement: Table S1 — (DOC) [file pone.0088914.s001.doc]

Table S1. Model results for land transformation at the string scale. Sum of variable AICc weights are: Land cover = 0.99, Topography = 0.46, Turbine size = 0.17, Configuration = 0.01.

| **Candidate models** | **K** | **AICc** | **Delta AICc** | **Model Likelihood** | **AICc Weight** | **Log Likelihood** | **Cumulative Weight** |
| --- | --- | --- | --- | --- | --- | --- | --- |
| Land cover | 6 | 717.62 | 0.00 | 1.00 | 0.43 | -351.50 | 0.43 |
| Land cover, Topography | 9 | 717.80 | 0.18 | 0.92 | 0.39 | -346.80 | 0.82 |
| Land cover, Turbine size | 7 | 720.52 | 2.89 | 0.24 | 0.10 | -351.45 | 0.92 |
| Land cover, Topography, Turbine size | 10 | 721.41 | 3.79 | 0.15 | 0.06 | -346.78 | 0.99 |
| Land cover, Configuration | 9 | 725.68 | 8.05 | 0.02 | 0.01 | -350.74 | 1.00 |
| Land cover, Configuration, Turbine size | 10 | 729.02 | 11.39 | 0.00 | 0.00 | -350.58 | 1.00 |
| Land cover, Topography, Configuration | 12 | 729.06 | 11.44 | 0.00 | 0.00 | -346.53 | 1.00 |
| Topography | 5 | 731.74 | 14.12 | 0.00 | 0.00 | -359.96 | 1.00 |
| Land cover, Topography, Configuration, Turbine size | 13 | 733.19 | 15.56 | 0.00 | 0.00 | -346.31 | 1.00 |
| Topography, Turbine size | 6 | 734.30 | 16.68 | 0.00 | 0.00 | -359.84 | 1.00 |
| Intercept only | 2 | 739.65 | 22.03 | 0.00 | 0.00 | -367.66 | 1.00 |
| Topography, Configuration | 8 | 739.98 | 22.35 | 0.00 | 0.00 | -359.59 | 1.00 |
| Configuration | 5 | 741.11 | 23.49 | 0.00 | 0.00 | -364.65 | 1.00 |
| Turbine size | 3 | 742.01 | 24.38 | 0.00 | 0.00 | -367.66 | 1.00 |
| Topography, Configuration, Turbine size | 9 | 742.63 | 25.00 | 0.00 | 0.00 | -359.21 | 1.00 |
| Configuration, Turbine size | 6 | 743.66 | 26.04 | 0.00 | 0.00 | -364.52 | 1.00 |
