# Supplementary material for: Land Cover and Topography Affect the Land Transformation Caused by Wind Facilities
Source: PLoS One. 2014 Feb 18;9(2):e88914. doi: 10.1371/journal.pone.0088914 (PMC3928332; doi:10.1371/journal.pone.0088914)
Supplement: Table S2 — (DOC) [file pone.0088914.s002.doc]

Table S2. Model results for land transformation at the site scale. Sum of AICc weights are: Land cover = 0.99, Topography = 0.74, Turbine size = 0.18, Configuration = 0.02.

| **Candidate models** | **K** | **AICc** | **Delta AICc** | **Model Likelihood** | **AICc Weight** | **Log Likelihood** | **Cumulative Weight** |
| --- | --- | --- | --- | --- | --- | --- | --- |
| Land cover, Topography | 9 | 735.71 | 0.00 | 1.00 | 0.63 | -355.75 | 0.63 |
| Land cover | 6 | 738.26 | 2.56 | 0.28 | 0.17 | -361.82 | 0.80 |
| Land cover, Topography, Turbine size | 10 | 739.14 | 3.43 | 0.18 | 0.11 | -355.64 | 0.91 |
| Land cover, Turbine size | 7 | 740.32 | 4.61 | 0.10 | 0.06 | -361.35 | 0.97 |
| Land cover, Configuration | 9 | 743.80 | 8.09 | 0.02 | 0.01 | -359.80 | 0.99 |
| Land cover, Configuration, Turbine size | 10 | 744.33 | 8.62 | 0.01 | 0.01 | -358.24 | 0.99 |
| Land cover, Topography, Configuration | 12 | 745.71 | 10.01 | 0.01 | 0.00 | -354.86 | 1.00 |
| Topography | 5 | 749.14 | 13.43 | 0.00 | 0.00 | -368.66 | 1.00 |
| Land cover, Topography, Configuration, Turbine size | 13 | 749.37 | 13.66 | 0.00 | 0.00 | -354.40 | 1.00 |
| Topography, Turbine size | 6 | 751.82 | 16.11 | 0.00 | 0.00 | -368.60 | 1.00 |
| Topography, Configuration | 8 | 757.17 | 21.46 | 0.00 | 0.00 | -368.18 | 1.00 |
| Topography, Configuration, Turbine size | 9 | 759.82 | 24.12 | 0.00 | 0.00 | -367.81 | 1.00 |
| Intercept, only | 2 | 762.13 | 26.42 | 0.00 | 0.00 | -378.90 | 1.00 |
| Turbine size | 3 | 764.36 | 28.66 | 0.00 | 0.00 | -378.84 | 1.00 |
| Configuration | 5 | 764.54 | 28.83 | 0.00 | 0.00 | -376.36 | 1.00 |
| Configuration, Turbine size | 6 | 767.34 | 31.63 | 0.00 | 0.00 | -376.36 | 1.00 |
