# Supplementary material for: Land Cover and Topography Affect the Land Transformation Caused by Wind Facilities
Source: PLoS One. 2014 Feb 18;9(2):e88914. doi: 10.1371/journal.pone.0088914 (PMC3928332; doi:10.1371/journal.pone.0088914)
Supplement: Table S4 — (DOC) [file pone.0088914.s004.doc]

Table S4. Model results for the mean nearest neighbor distance between turbines. Sum of AICc weights are: Land cover = 0.97, Topography = 0.03, Turbine size = 0.99, Configuration = 0.05.

| **Candidate models** | **K** | **AICc** | **Delta AICc** | **Model Likelihood** | **AICc Weight** | **Log Likelihood** | **Cumulative Weight** |
| --- | --- | --- | --- | --- | --- | --- | --- |
| Land Cover, Turbine Size | 7 | 463.65 | 0.00 | 1.00 | 0.93 | -223.02 | 0.93 |
| Land Cover, Configuration, Turbine size | 10 | 470.50 | 6.86 | 0.03 | 0.03 | -221.32 | 0.96 |
| Topography, Configuration, Turbine Size | 9 | 471.75 | 8.10 | 0.02 | 0.02 | -223.77 | 0.98 |
| Land Cover, Topography, Turbine Size | 10 | 473.45 | 9.81 | 0.01 | 0.01 | -222.80 | 0.99 |
| Turbine Size | 3 | 473.47 | 9.83 | 0.01 | 0.01 | -233.39 | 0.99 |
| Topography, Turbine Size | 6 | 475.02 | 11.37 | 0.00 | 0.00 | -230.20 | 1.00 |
| Configuration, Turbine Size | 6 | 475.68 | 12.03 | 0.00 | 0.00 | -230.53 | 1.00 |
| Land Cover | 6 | 476.59 | 12.94 | 0.00 | 0.00 | -230.98 | 1.00 |
| Land cover, Topography, Configuration, Turbine size | 13 | 479.75 | 16.10 | 0.00 | 0.00 | -219.59 | 1.00 |
| Intercept, Only | 2 | 480.95 | 17.31 | 0.00 | 0.00 | -238.31 | 1.00 |
| Land Cover, Configuration | 9 | 483.40 | 19.75 | 0.00 | 0.00 | -229.59 | 1.00 |
| Topography | 5 | 484.11 | 20.47 | 0.00 | 0.00 | -236.15 | 1.00 |
| Land Cover, Topography | 9 | 485.33 | 21.68 | 0.00 | 0.00 | -230.56 | 1.00 |
| Configuration | 5 | 487.79 | 24.15 | 0.00 | 0.00 | -237.99 | 1.00 |
| Topography, Configuration | 8 | 490.98 | 27.34 | 0.00 | 0.00 | -235.09 | 1.00 |
| Land Cover, Topography, Configuration | 12 | 494.73 | 31.09 | 0.00 | 0.00 | -229.37 | 1.00 |
